# Interaction and verification of ferroptosis-related RNAs Rela and Stat3 in promoting sepsis-associated acute kidney injury

**DOI:** 10.1515/med-2025-1156

**Published:** 2025-04-02

**Authors:** Yang Cao, Yansong Liu, Yunlong Li, Junbo Zheng, Yue Wang, Hongliang Wang

**Affiliations:** Department of Intensive Care Medicine, The Second Affiliated Hospital of Harbin Medical University, Harbin, 150001, Heilongjiang, China; Department of Pharmacology & Toxicology, Wright State University, Dayton, United States of America; Department of Intensive Care Medicine, The Second Affiliated Hospital of Harbin Medical University, No. 246 Xuefu Road, Nangang District, Harbin, 150001, Heilongjiang, China

**Keywords:** sepsis, AKI, transcriptomics, ferroptosis-related RNA, prognosis

## Abstract

**Background:**

Sepsis is a prevalent and severe condition. However, research investigating the relationship between the immune microenvironment in sepsis-associated acute kidney injury (SA-AKI) through diagnostic models using RNA biomarkers remains limited. Therefore, this study developed a diagnostic model using gene expression data from the Gene Expression Omnibus (GEO) database, leveraging a sufficient sample size.

**Methods:**

We proposed a computational method to identify RNAs Rela and Stat3 constructing a diagnostic model using Least Absolute Shrinkage and Selection Operator regression algorithms. Gene expression data from the GEO, comprising five samples each of SA-AKI and sepsis, were analyzed.

**Results:**

Diagnostic models were developed for the datasets, followed by immune cell infiltration and correlation analyses. Experiments were conducted to test and confirm the high expression of Stat3 via Rela in AKI cells post-sepsis, leading to a worse prognosis.

**Conclusion:**

This study identified the significant roles of RNAs Rela and Stat3 in SA-AKI. The developed diagnostic model demonstrated improved accuracy in identifying SA-AKI, suggesting that these RNA markers may provide valuable insights into the pathophysiology of SA-AKI and enhance early diagnosis. These findings contribute to a better understanding of immune-related mechanisms underlying SA-AKI and may inform future therapeutic strategies.

## 1 Introduction

Sepsis is the leading cause of infection-related death globally, posing a significant global health threat [1,2,3]. Early recognition and diagnosis are crucial to prevent the progression of septic shock, which has a mortality rate of ≥40% [4]. Research indicates that host genetic variants significantly affect sepsis susceptibility. However, the results are inconsistent [5]. The international expert consensus identified patient heterogeneity as a factor for clinical trial failures in sepsis treatment [6,7], highlighting the need for reliable markers to identify high-risk patients for detection and prevention.

Sepsis-associated acute kidney injury (SA-AKI) is a renal failure caused by sepsis [8,9], a leading cause of AKI, with heightened risk among affected patients. The incidence of SA-AKI varies by region and population, particularly in ICU settings, the [10,11,12] mortality rate ranges from 30 to 70%. The mortality rate is higher in patients with SA-AKI than in those with sepsis without AKI [13,14]. SA-AKI has a high incidence and mortality. Early diagnosis, aggressive treatment, and effective renal support measures can improve patient prognosis. However, the mechanism underlying SA-AKI remains under-researched.

Reportedly, adult adoptees whose biological parents died from an infection before age 50 have a 5.81 times increased risk of dying from infection, surpassing the relative risk of dying from cardiovascular disease or cancer. This reveals, for the first time, the significant effect of host genetics on infectious disease development [15,16,17]. Subsequent studies increasingly emphasize the significant role of genetic variants in determining individual differences in patients with sepsis clinical outcomes and inflammatory responses [18,19].

Microarray technology, along with advancements in transcriptome technology, has been extensively used to investigate disease mechanisms [20,21,22,23]. The microarray and RNA-sequencing technologies data are analyzed separately, considering their differences [24,25].

Building machine-learning models based on RNA expression data encounters challenges in identifying the most informative index or features for classification. To address this issue, different machine learning methods such as Least Absolute Shrinkage and Selection Operator (LASSO) and Support Vector Machine (SVM), have been developed [24,26,27]. These methods assist in classifying gene data, diagnosing diseases, studying cell migration and microbiomes, and significantly enhance feature learning owing to their high classification accuracy, whether used individually or in combination [28,29]. Therefore, this study aims to investigate the development of a sepsis-specific diagnosis using a combination of LASSO and SVM algorithms, employing a publicly available database. Initially, the LASSO classifier was used to identify essential genes for classification. Subsequently, SVM was employed to calculate the weights of these essential genes in two separate datasets. Finally, a scoring model for neural sepsis was developed by integrating LASSO and SVM. Microarray and RNA-seq data were utilized to evaluate the accuracy and superiority of the constructed diagnostic model and evaluate its performance. First, a differential analysis was conducted on SA-AKI models related to ferroptosis, and a prediction model was constructed. The analysis revealed higher expression of Rela in sepsis-associated AKI than in sepsis samples without AKI. The protein-level expression difference of Rela was verified. Enrichment analysis revealed a strong correlation index between Rela and Stat3 in SA-AKI. Knocking down Rela expression in two SA-AKI cell types revealed a corresponding decrease in Stat3 in SA-AKI, which decreased accordingly. It proved that Stat3 expression was present, confirming a positive correlation between Stat3 and Rela in SA-AKI.

## 2 Methods and materials

### 2.1 Research design

LASSO and SVM were used to establish the sepsis diagnosis model. The GSE60088 dataset (*n* = 10) was used to screen differentially expressed genes (DEGs), followed by gene enrichment analysis to identify pivotal genes for classification. LASSO regression in RNA-seq was used to calculate gene weights and develop a classification model. Western blot (WB) was conducted to verify higher Rela expression in SA-AKI cells, with sepsis induction via Stat3 for further validation.

### 2.2 Data selection and analysis

An extensive search was conducted on the Gene Expression Omnibus (GEO) database using the keywords “sepsis,” “Homo sapiens,” and “AKI.” One RNA-seq dataset with 10 samples was downloaded from the GEO database and converted to a logarithmic form, with batch effects removed using the R package ComBat.

### 2.3 DEGs screening

The GSE60088 dataset obtained via the Affymetrix Human Genome Array included five sepsis and five control cases, which were used for DEG analysis. The limma package in R was used to calculate the two groups based on the classical Bayesian method with a significance threshold of *P* < 0.01 and an absolute value of |log fold change| >1. Additionally, heat maps and corrplots were generated for data visualization.

### 2.4 Enrichment analysis

Enrichment analysis was further used to confirm the biological function of selected DEGs using the Kyoto Encyclopedia of Genes and Genomes (KEGG) and Gene Ontology (GO). Significant enrichment terms meeting the threshold *P* < 0.01 under the Benjamini and Hochberg adjustment were screened. A heatmap was created for genes with an absolute value of essential degree >0.2.

### 2.5 LASSO classification

LASSO is a popular machine-learning algorithm used for regression and classification tasks. The algorithm selects the most relevant features while reducing model complexity. The LASSO algorithm achieves feature selection and model regularization through the coefficient constraint of each feature. Specifically, the algorithm uses L1 regularization to control model complexity and balance fitting and generalization abilities by adjusting the regularization parameter *λ*. As the *λ* value increases in the LASSO algorithm, the coefficients of most features decrease to zero, retaining only essential features. Compared to other regularization techniques, the LASSO algorithm has several advantages: it handles high-dimensional data effectively, even when sample numbers are less than the feature numbers, and performs simultaneous feature selection and model regularization, thereby enhancing model interpretability and generalizability. It can reduce model overfitting and improve robustness.

Therefore, the Lasso algorithm is extensively applied in various fields, such as bioinformatics and image processing.

### 2.6 SVM classification

The SVM algorithm was implemented. Data preprocessing involved cleaning, denoising, and normalizing to enhance classification suitability. Raw data were converted into feature vectors for model training using techniques such as the bag-of-words model or term frequency-inverse document frequency. SVM was used to maximize the classifier margin by solving a convex quadratic programming problem, using methods such as sequential minimal optimization algorithm or quadratic programming solver. Cross-validation and other techniques were used to evaluate and tune the parameters for optimal performance and generalization ability. The trained model was used to classify new test samples and make predictions accordingly.

### 2.7 Gene set enrichment analysis (GSEA)

The expression profile data was subjected to quality control, denoising, and normalization preprocessing. Gene sets relevant to the research objective, such as pathways or functional annotations from databases, including KEGG or GO, were selected. Enrichment scores for each gene set were calculated by comparing their gene expression values in the samples to randomly permuted gene expression values. Statistical significance testing was conducted using permutation tests, simulations, or empirical distribution analysis methods to assess the reliability of the results from GSEA. The results were interpreted to reveal changes in specific biological processes or signaling pathways under different conditions.

### 2.8 Area under curve (AUC) performance evaluation

The dataset was divided into training and testing sets. The training set was used to train the classifier model, while the testing set was used to evaluate its performance. The selected classification algorithm was used to train the training set, resulting in a trained classifier model. The testing set was used to evaluate the performance of the classifier, with the AUC value calculated as a measure of classifier performance. Notably, the AUC value was obtained by plotting the receiver operative characteristics (ROC) curve and computing the area under the ROC curve. Multiple classifier models were compared to identify the better performers. Statistical significance tests, such as the Wilcoxon–Mann–Whitney *U*-test or confidence interval comparisons of two AUC values, were used. The performance of each classifier was evaluated and compared based on the AUC value and statistical analysis results.

### 2.9 Cell culture and transfection

The human renal tubular epithelial cell line HK2 was obtained from the Cell Bank of the Chinese Academy of Sciences (Shanghai, CHN). RPMI 1640 medium supplemented with 10% FBS and incubated at 5% CO_2_ and 37°C was utilized. Lipofectamine™ 3000 (Invitrogen, Carlsbad, CA, US) was used to transfect HK2 shRela cells with Rela shRNA and negative shRNA vectors, following the protocols of the manufacturer. The effective knockdown of the target gene by shRNA was demonstrated through quantitative RT-PCR and WB analyses measuring alterations at the mRNA and protein levels. Results indicated a significant reduction in mRNA levels and corresponding protein expression in the shRNA-treated group compared with controls, suggesting successful gene knockdown by shRNA. To exclude off-target effects of shRNA, three independent shRNA sequences were designed, all confirming effective knockdown of the target gene expression. The shRNA sequence is presented in Table 1.

**Table 1:** Design of shRNA sequences targeting Rela gene and knockdown validation in HK2 cells

No.	5′	STEM	Loop	STEM	3′
Rela-RNAi(127815-1)-a	Ccgg	GAAGACATTGAGGTGTATTTC	CTCGAG	GAAATACACCTCAATGTCTTC	TTTTTg
Rela-RNAi(127815-1)-b	aattcaaaaa	GAAGACATTGAGGTGTATTTC	CTCGAG	GAAATACACCTCAATGTCTTC	
Rela-RNAi(127816-1)-a	Ccgg	GGACCTATGAGACCTTCAAGA	CTCGAG	TCTTGAAGGTCTCATAGGTCC	TTTTTg
Rela-RNAi(127816-1)-b	aattcaaaaa	GGACCTATGAGACCTTCAAGA	CTCGAG	TCTTGAAGGTCTCATAGGTCC	
Rela-RNAi(127817-2)-a	Ccgg	GCAGTTTGATGCTGATGAAGA	CTCGAG	TCTTCATCAGCATCAAACTGC	TTTTTg
Rela-RNAi(127817-2)-b	aattcaaaaa	GCAGTTTGATGCTGATGAAGA	CTCGAG	TCTTCATCAGCATCAAACTGC	

### 2.10 WB analysis

The total proteins were separated using 10% sodium dodecyl sulfate-polyacrylamide gel electrophoresis and transferred onto nitrocellulose membranes. After blocking in 5% phosphate-buffered saline with Tween 20 (PBS-T) for 2 h, the membranes were incubated overnight at 4°C with primary antibodies and for 2 h at room temperature with secondary antibodies. PBS-T was used to rinse the membranes thrice at intervals during the incubation. Finally, specific protein blots were visualized following the instructions of the manufacturer. The membrane-washing solution was drained. Subsequently, the membranes were protein-bound upward, and the prepared ECL working solution (Meilunbio, China) was added. During incubation, we ensured the working solution covered the entire membrane, while incubation was conducted for 1–2 min at room temperature. The processed film was placed in the X-ray cassette for exposure and development. Blots were cut before hybridization with antibodies to avoid non-specific band staining. The marker positions were aligned according to the protocol instructions for protein band detection. For further experiments, the membrane was cut approximately 30 kDa above and below the protein bands for antibody incubation. Protein band analysis was conducted using ImageJ software.

### 2.11 Construction of the SA-AKI cell model

In the lipopolysaccharide (LPS) group, 15 µg/mL LPS was added to the culture medium and incubated for 4–24 h. In the cisplatin group, 25 µM cisplatin was added to the culture medium and incubated for 24–48 h. The control group was treated with an equal volume of PBS-T as a reagent blank control. SA-AKI was defined as occurring when over half of the experimental group cells exhibited morphologically evident damage compared with the control group. This procedure was repeated three times.

### 2.12 Statistical analysis

All experiments were replicated at least three times to ensure result reliability. Sample size determination was based on prior statistical power analysis, ensuring adequate statistical power to detect biologically significant differences. Additionally, comprehensive statistical reports were provided to substantiate our conclusions. All statistical analyses were conducted using R software version 4.0.2. Kaplan‒Meier analysis and log-rank tests were used to evaluate survival and compare differences between clusters and risk groups. A significance level of *P* < 0.05 was applied.


**Informed consent:** GEO is a public database, and all patient-related data have received ethical clearance. Users can freely download relevant information for study and publication purposes.
**Ethical approval:** We acknowledge the GEO and TCGA databases for providing a platform where contributors upload valuable datasets. Our research is according to open-source information, thus avoiding ethical issues and conflicts of interest.

## 3 Results

### 3.1 Identification of DEGs

**Figure 1:**
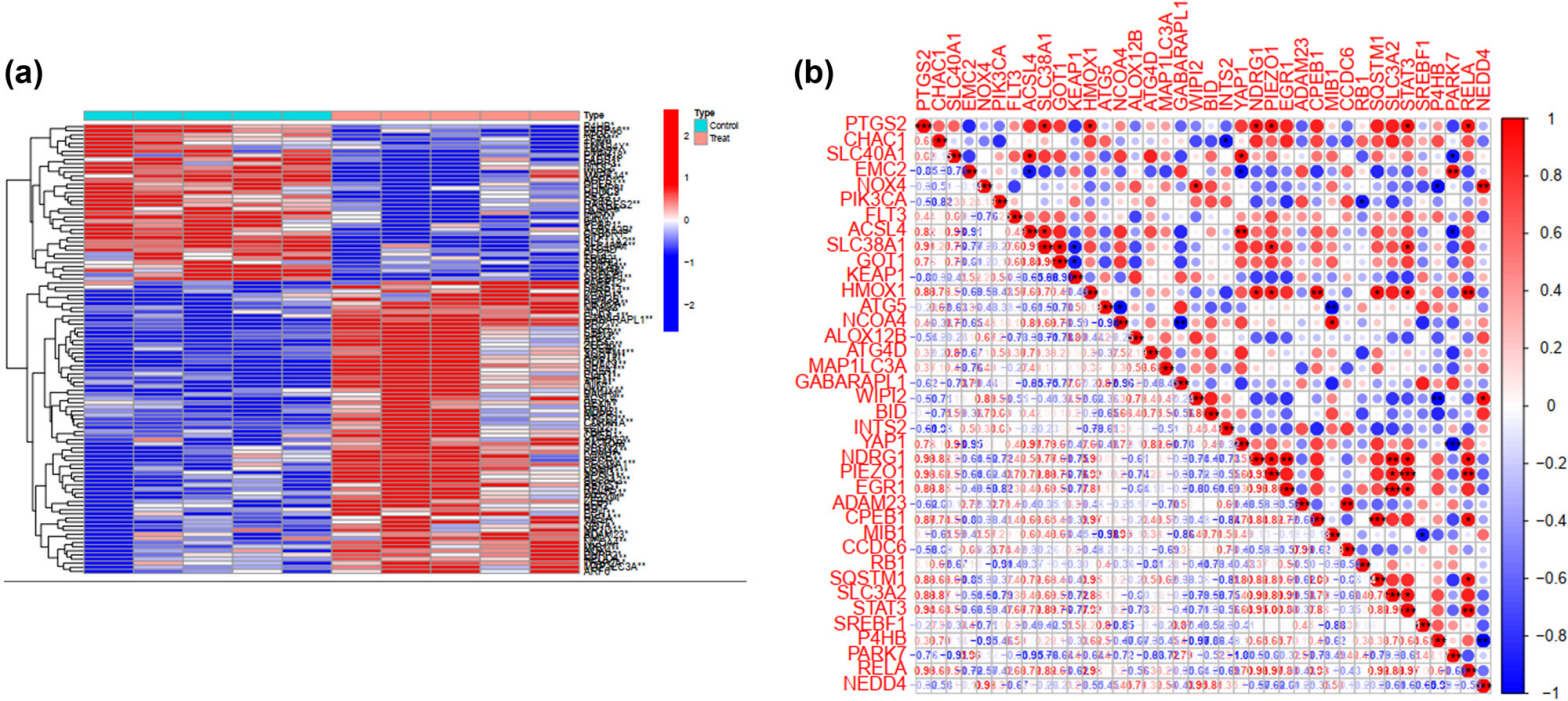
(a) 100 DEGs in the training data set. Each row and column represent a gene and sample, respectively. Red and blue colors indicate higher and lower expression levels, respectively. The left and right sides represent the results of the control and experimental groups, respectively (*P* < 0.01, |logFC| > 1). (b) Analysis and interpretation of the correlation between the variables, based on the generated visualization, considering the direction and strength of positive or negative correlations as well as the significance level of the correlation coefficient. The two selected key sepsis-associated acute kidney injury (SA-AKI) genes (i.e., Rela and Sta3) exhibited high correlation.

The heatmap shows RNA expression levels in the dataset (Figure 1a). After annotation, 18,501 gene symbols were identified, and a heatmap plot was used to represent the DEG distribution, comprising 50 upregulated and 50 downregulated genes. The red splash denotes significantly upregulated genes, while blue indicates downregulated genes. White splashes represent genes with no significant difference in expression. Figure 1b displays the corrplot map for 100 DEGs in the training dataset. The “corrplot” R package was used to generate a visual representation of the correlation matrix. Various parameters, such as color, font size, and line width, were adjusted to clarify the displayed correlation information. The results showed a high correlation between Rela and Stat3 in SA-AKI cells (Figure 1b).

### 3.2 Enrichment analysis

**Figure 2:**
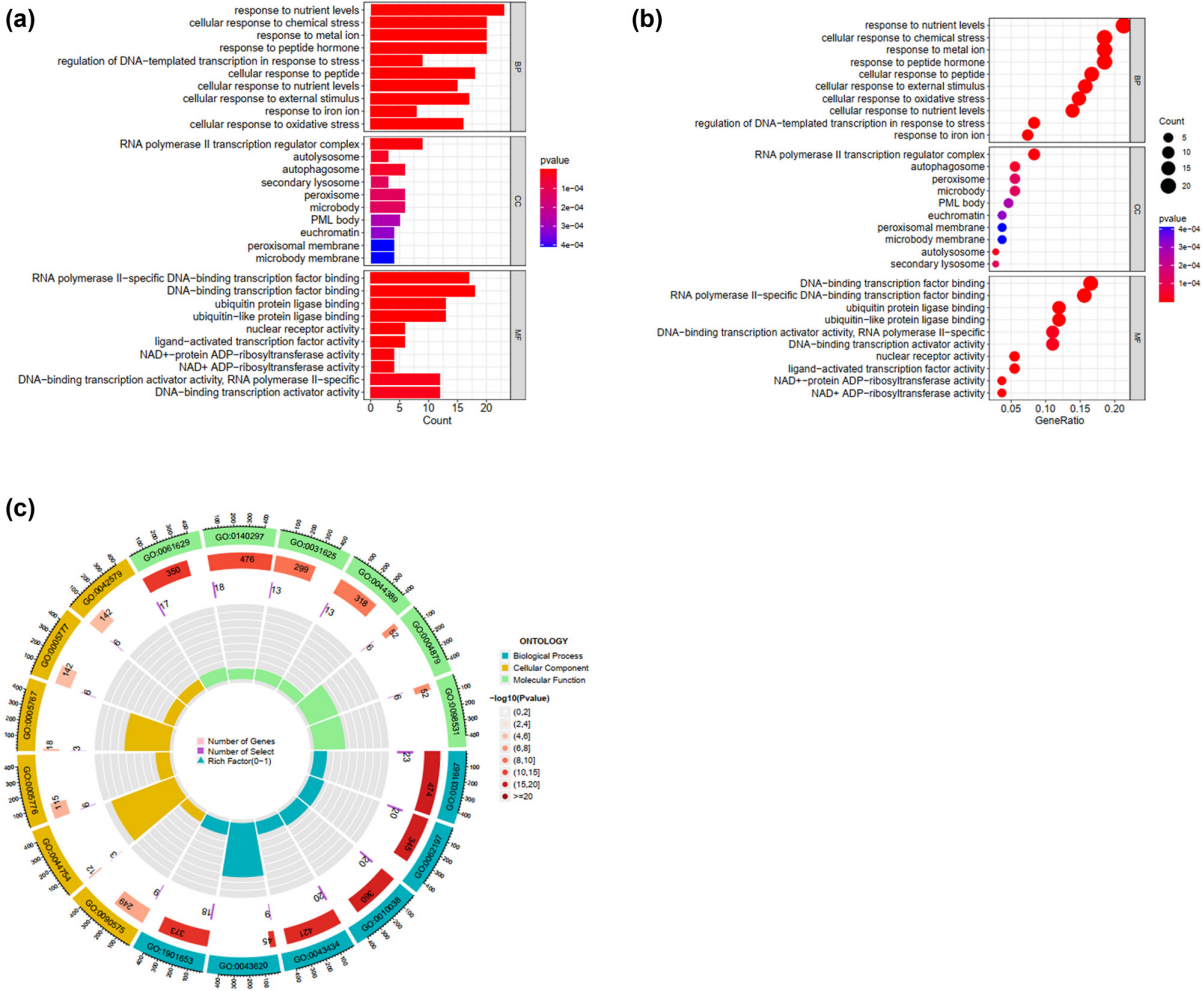
Gene ontology (GO) enrichment analysis around the key genes Rela and Stat3. (a) Coessential analysis of Rela for the SA-AKI models. (b) Bubble plot of the gene enrichment analysis, indicating high essential degrees of ferroptosis and iron ions. (c) GO circle plot for the gene enrichment analysis from panel B.

**Figure 3:**
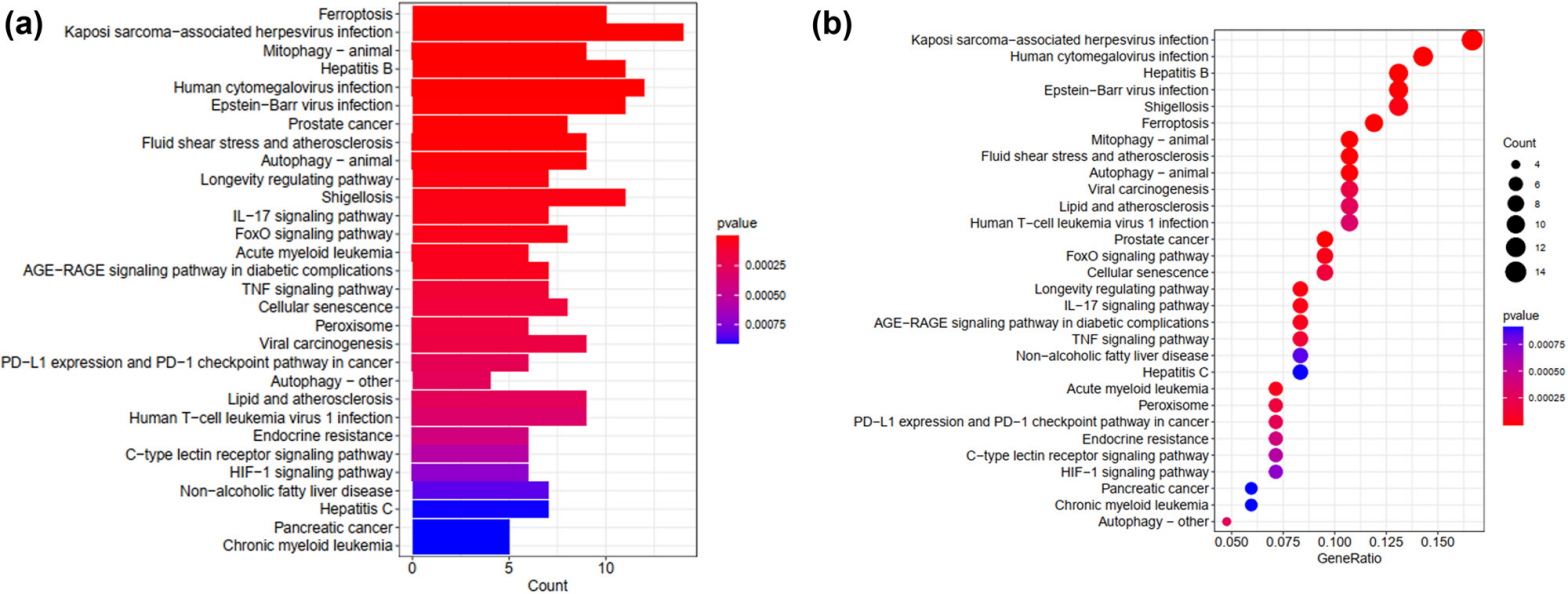
Kyoto Encyclopedia of Genes and Genomes enrichment analysis around the key genes Rela and Stat3. (a) Coessential analysis of Rela for the SA-AKI models. (b) Bubble plot of the gene enrichment analysis, indicating high essential degrees of ferroptosis.

Enrichment analysis was used to confirm the biological function of the selected gene Rela (Figure 2a) using GO analysis. Significant enrichment terms with a threshold of *P* < 0.01, adjusted using the Benjamini and Hochberg method, were screened (Figure 2c). Additionally, a bubble plot was generated for genes with an essential degree absolute value of >0.1 in SA-AKI models (Figure 2b). First, the database was assessed to select the stomach tissue, gastric cancer cell lines, and TP73 from associated genes. The column in Figure 2a presents enriched functional pathways, while Figure 2b depicts a bubble plot of gene enrichment highly correlated with Rela. The closer to red, the higher the positive correlation; the closer to blue, the higher the negative correlation. Figure 2c shows the GO circle plot pathway obtained from the enrichment analysis of these highly correlated genes. Furthermore, statistical analysis on the pathways enriched by DEGs was conducted using the clusterProfiler enrichment analysis software. Multiple testing corrections, including FDR and other methods, were applied to determine the significance level of the pathways. KEGG diagrams were plotted to represent the DEGs between the experimental and control groups, employing color, line thickness, and other visualization methods. Figure 3b shows the enrichment and significance levels of each pathway. The generated visualization showed a high correlation between ferroptosis and DEGs (Figure 3b).

### 3.3 Screening candidate and validation of SA-AKI-Specific genes using LASSO and SVM

**Figure 4:**
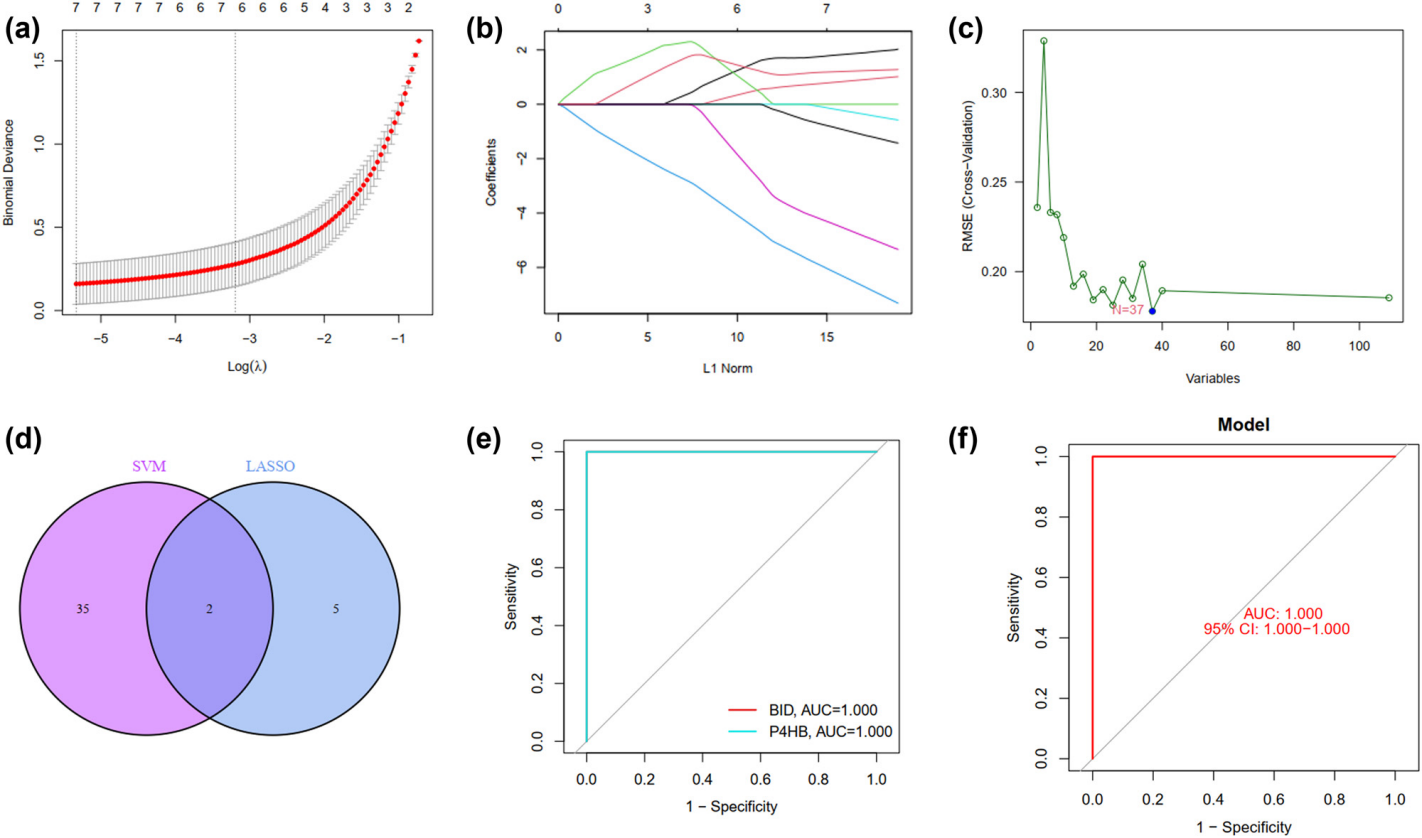
(a)–(c) Network of topology came from the LASSO and SVM for the dataset. (d) The Venn diagram represents the genes selected by taking the intersection of the LASSO and SVM models. (e) This figure represents the sensitivity and specificity of the BH3 Interacting Domain Death Agonist and Prolyl 4-Hydroxylase Subunit Beta model, which was built based on the intersection genes, in distinguishing SA-AKI. (f) Performance evaluation of sepsis by the area of the receiver operating characteristic curve calculated and their AUC values for the dataset validation data.

The 100 obtained DEGs were inputted into LASSO (Figure 4a and b) and SVM (Figure 4c) classifiers to obtain sepsis-specific genes. A Venn diagram shows that BH3 Interacting Domain Death Agonist and Prolyl 4-Hydroxylase Subunit Beta (P4HB) were core genes in the algorithms (Figure 4d). The DEG-induced models for predicting SA-AKI occurrence demonstrated satisfactory accuracy in sensitivity and specificity, as calculated using ROC and AUC (Figure 4e and f).

### 3.4 GSEA of Stat3-associated network

**Figure 5:**
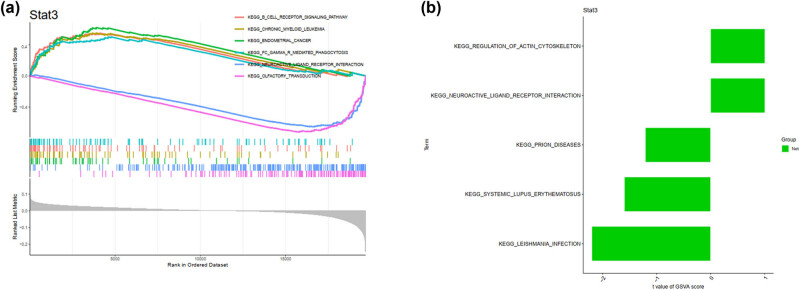
Performance evaluation of Stat3 in the SA-AKI models by GSEA. (a) GSEA was performed to identify enriched KEGG pathways in the high- versus low-risk groups. The *X*- and *Y*-axes represent the normalized enrichment score and the different Stat3-related KEGG pathways, respectively. (b) GSEA to assess the overall activity level of immune-related pathways between the two groups. The *X*- and *Y*-axes represent the sample score and the pathway, respectively. The *P*-value was calculated using the Wilcoxon rank-sum test, and FDR correction was applied. Error bars represent the standard errors of the mean.

clusterProfiler was used for statistical analysis of pathways enriched by DEGs. Multiple testing corrections were conducted using FDR or other methods to determine the significance level of the pathways. GSEA diagrams were plotted to display differential gene expression between experimental and control groups using color, line thickness, and other methods. The enrichment and significance levels of each pathway were indicated in the diagram (Figure 5a). Based on the generated visualization, pathway differences between the experimental and control groups were analyzed to infer the biological functions of genes and their relationship with related diseases (Figure 5b). Statistical tests, such as the Kolmogorov–Smirnov and the Wilcoxon rank sum tests, were conducted to verify the effectiveness and robustness of the analysis results.

### 3.5 Immune cell infiltration and correlation analysis

**Figure 6:**
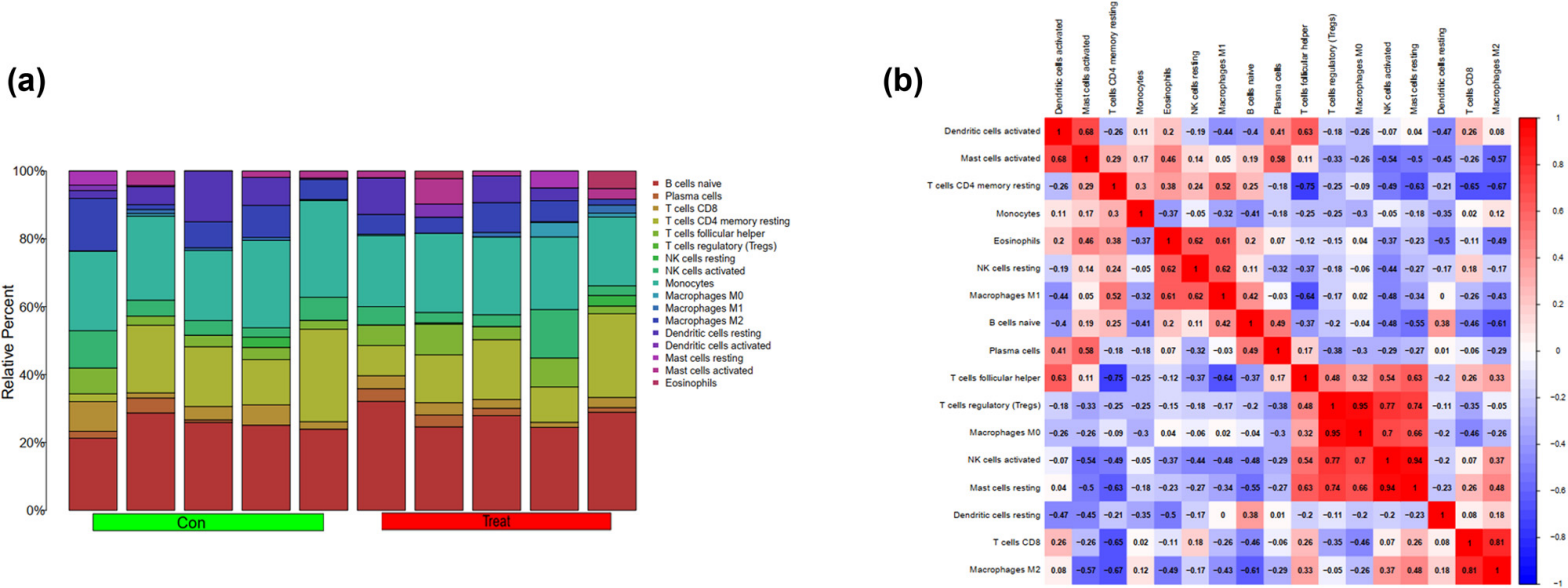
(a) The columns and colors represent different samples and different immune cell subtypes. The length of each cell represents the relative proportion of the immune cell subtype in that sample, with longer and shorter bars indicating higher and lower proportions, respectively. (b) Correlation matrix of different immune cell types. Each small square in the figure represents the correlation between two immune cell types, with the color and numerical value representing the degree of correlation. Different colors indicate different degrees of correlation, with red and blue representing strong positive and blue negative correlations, respectively. Based on these correlation data, further analysis could be conducted to explore the interactions between the different immune cell types and their influence.

Figure 6a shows the immune cell infiltration analysis in SA-AKI models, highlighting the interactions and regulatory mechanisms between different immune cell types. Analysis revealed complex interactions and regulatory relationships between different immune cell types. The CIBERSORT algorithm was used to extract 22 immune cell subtypes from tumor tissue and calculate their relative proportions in different samples. The results were displayed using a color legend to show differences. Each small square represents the relationship between a specific immune cell type and a tissue area, with the color indicating the relative quantity or expression level of that immune cell type. The results showed increased regulatory T cells, macrophages, and natural killer (NK) cells and reduced CD8+ T cells and eosinophils in all samples. Figure 6b displays a network diagram of the correlations and functional modules between different immune cell types. The correlation data were processed and analyzed to establish and visualize an immune cell interaction network. In this figure, each node represents an immune cell type, with different colors representing different functional modules, while the color of the module indicates interaction strength. The figure illustrates the relationships between various immune cells, including B cells (B) and T cells (T), which are lymphocytes involved in antibody-mediated immune responses. NK cells can kill infected cells and regulate the activity of other immune cells. Dendritic cells activated T and B cells as specialized antigen-presenting cells. Eosinophils and basophils participated in immune responses related to sepsis. The figure shows close interactions between T, B, and NK cells, indicating common functions and regulatory mechanisms in antigen presentation and antibody production related to sepsis.

### 3.6 Drug sensitivity analysis

**Figure 7:**
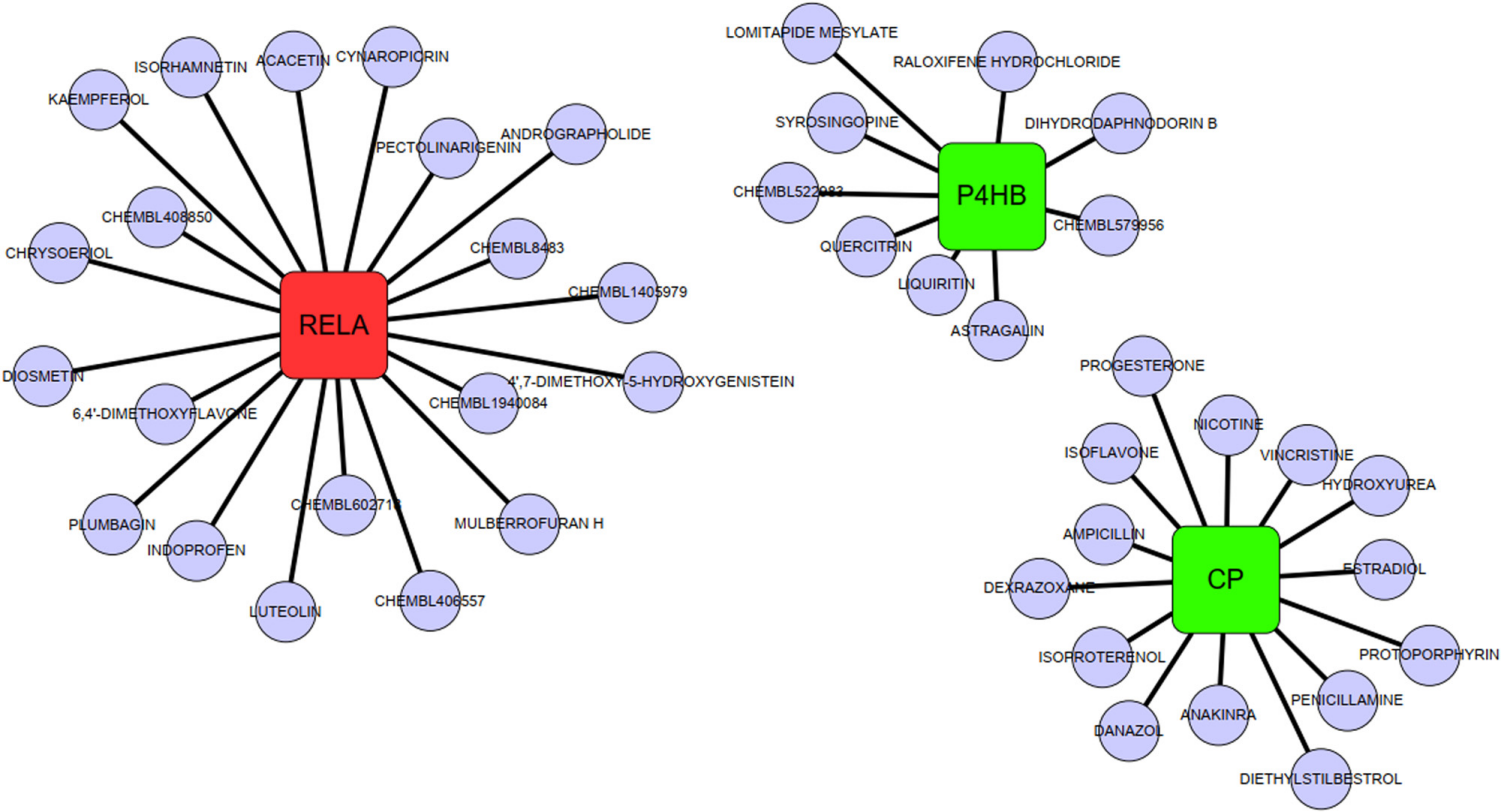
Visualization of the Drug Gene Interaction Database drug sensitivity analysis. Based on the obtained results, drug sensitivity curves and heat maps were plotted to indicate the impact of the drugs on different genes using colors and line thicknesses.

Drug-gene interaction data was obtained from the Drug Gene Interaction Database, and drugs for sensitivity analysis were selected. LASSO regression was used to analyze Stat3, CP, and P4HB gene expression in SA-AKI models to evaluate drug sensitivity differences. Dexamethasone showed the highest sensitivity for the Rela gene in SA-AKI (Figure 7), as shown in the graph. A *t*-test was conducted to verify the effectiveness and robustness of the results.

### 3.7 HK2 cells exhibit high expression of Stat3 via Rela

**Figure 8:**
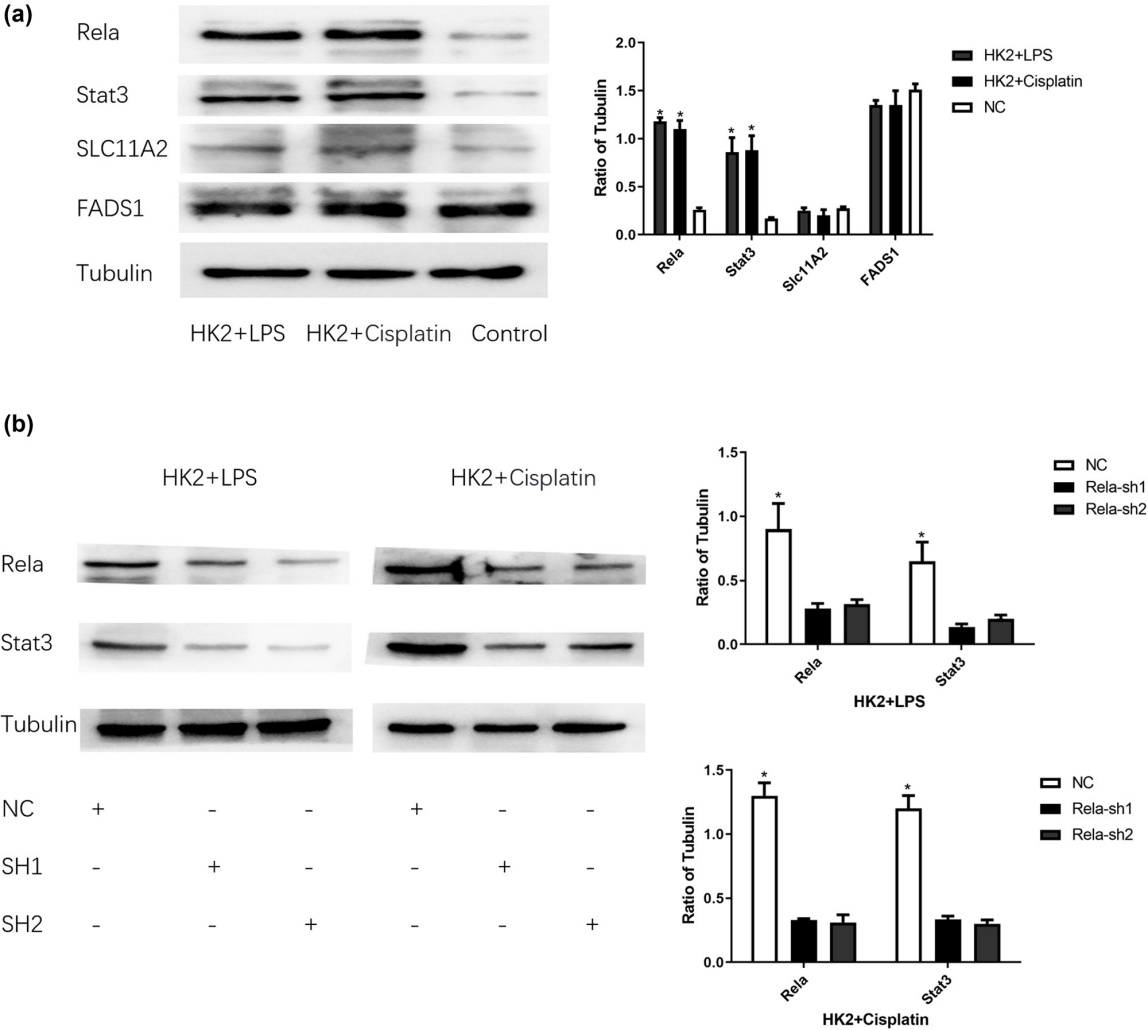
(a) Rela, Stat3, Slc11a2, and Fads1 protein expressions in SA-AKI and normal HK2 cells singly. *P < 0.05. (b) Rela and Stat3 protein expressions after Rela silencing. *P < 0.05.

The WB experiment revealed a significant increase in Stat3 expression in HK2 cells after sepsis (Figure 8a). Cluster analysis showed a close association between Stat3 expression in HK2 cells and Rela. Knocking down Rela expression using shRNARela resulted in a significant reduction in Stat3 expression (Figure 8b).

## 4 Discussion

Rapid advancements in machine learning algorithms and the availability of gene expression data in public databases have significantly improved the inference of biomarkers for disease diagnosis and prognosis in relevant fields [30,31,32,33]. Researchers have explored the use of different machine-learning algorithms to enhance sepsis diagnosis [34,35]. This study developed a diagnostic model using gene expression data from the GEO database, leveraging a sufficient sample size. The LASSO and SVM algorithms were combined to obtain essential classification genes and calculate their weights.

The GSE60088 was used to identify DEGs, excluding those with low expression levels to focus on practical genes. Enrichment analysis was conducted, and the results were visualized using bar and bubble plots. From the 30 enriched terms, four relevant terms were identified: neutrophil degranulation, neutrophil activation in immune response, specific granule activity, and immune receptor activity. The MeanDecreaseGini method was used to obtain the Top 2 core genes for classifying DEGs. Additionally, four out of the ten genes – Rela, Stat3, Slc11a2, and Fads1 – were identified as candidate genes for sepsis in previous studies. The diagnostic model incorporated the crucial genes crucial for classification with their respective weights, demonstrating its novelty. To validate the applicability and superiority of the model over others, WB was conducted. This confirmed higher Rela expression in human renal tubular epithelial cells post-sepsis via Stat3, inducing ferroptosis and a poorer diagnosis. Experimental findings suggest a pivotal role for ferroptosis in SA-AKI pathogenesis. The administration of ferroptosis inhibitors notably attenuated renal injury in SA-AKI models. Furthermore, shRNA-mediated knockdown of ferroptosis-associated genes reduced the severity of renal damage. These outcomes underscore ferroptosis as a cardinal pathogenic mechanism in SA-AKI.

The enrichment pathways of genes highly related to Rela in HK2 cells were analyzed. The KEGG results showed its primary involvement in stress response. Regarding cell composition, these genes are primarily involved in iron ion metabolism, which is highly related to ferroptosis. In biochemical reactions, these genes are primarily involved in ferroptosis pathways.

However, this study had some limitations. First, the study was conducted with a small sample size (PCOS: *n* = 5; normal: *n* = 5). Despite the removal of batch effects, the dataset was not optimal. The availability of public sequencing data for sepsis and AKI research is currently limited. The samples used in this research were obtained from the GSE60088 dataset available in the GEO public database, which is based on rat models developed for SA-AKI and control groups to acquire renal tissue for sequencing. This implies that our sequencing results may exhibit certain biases when compared with real-world scenarios, representing a significant limitation of our study. Several screening procedures were conducted, leading to the identification of only one relatively complete SA-AKI chip dataset suitable for investigation. Therefore, this modeling solely includes AUC data from the training group. Owing to insufficient data, transcriptome sequencing data post-sepsis of AKI chips for further validation were unavailable, resulting in the omission of test group verification. Therefore, future studies are needed to develop a single tissue-type diagnostic model using more comprehensive datasets and appropriate machine learning algorithms. Increasing sample size could facilitate the transition from observational research to prospective research, potentially enhancing the applicability of the model in clinical settings. Batch effects are inevitable for transcriptome data [36,37], with high-throughput batch effects having a more significant influence on the data [38,39,40]. To minimize this effect during data analysis, the data chips selected were all exclusively from the same laboratory, reducing systematic errors such as sampling and sequencing biases in the data analysis process. Second, while the potential impacts of age, race, gender, and other factors on the results remain unclear, we believe that using standardized animal models minimizes the interference of such systematic errors on the experimental outcomes. Additionally, it is extremely challenging to obtain renal tissues from patients with SA-AKI. Although we have made considerable efforts to collect samples, the current number of samples is insufficient to support further sequencing studies. Nevertheless, we will continue to strive to gather more samples, which will be a key focus of our future research. We acknowledge its limitations, including a modest sample size that restricts the generalizability of our findings and a focus primarily on these two RNAs, which may overlook other significant genes and pathways. Although we are aware of the importance of *in vivo* studies and controlling for confounding factors, our current study is constrained by resource limitations and a sample size that hinders a more comprehensive exploration. Additionally, our use of machine learning algorithms may raise concerns about overfitting, and our study lacks a detailed mechanistic understanding of the interaction between Rela and Stat3. We look forward to future research that can address these limitations and further elucidate the molecular mechanisms of SA-AKI. We hope that our findings can guide clinical diagnosis and treatment for SA-AKI; however, under the present circumstances, using rat models remains a relatively ideal research approach.

The construction of the sepsis model in HK2 cells revealed that high Rela expression partially represents HK2 cell sepsis. Protein level analysis in SA-AKI HK2 cells compared to normal epithelial cells demonstrated increased Rela expression in SA-AKI cells. Systemic AKI in patients with sepsis often triggers a strong immune response, potentially leading to kidney damage and prolonging patient prognosis. In contrast, patients with SA-AKI surviving sepsis typically exhibit enhanced physical condition compared to patients with sepsis only[13,41], thus correlating with a better prognosis.

Overall, the model demonstrates better performance in predicting and diagnosing sepsis, potentially enhancing the prognosis of patients with sepsis. These findings provide valuable evidence for future clinical applications in sepsis management.

In conclusion, this study identified candidate sepsis genes, Rela and Stat3, and developed a diagnostic model incorporating essential genes for comprehensive classification. Notably, higher expression of Stat3 was observed in HK2 cells post-sepsis through Rela, potentially inducing ferroptosis and a worse prognosis.

## 5 Conclusion

Candidate sepsis genes were identified in this study: Rela and Stat3. The diagnostic model considered essential genes for comprehensive classification.

Higher expression of Stat3 was observed in HK2 cells post-sepsis through Rela, potentially inducing ferroptosis and a worse prognosis.
